# Supplementary material for: Transient structural variations have strong effects on quantitative traits and reproductive isolation in fission yeast
Source: Nat Commun. 2017 Jan 24;8:14061. doi: 10.1038/ncomms14061 (PMC5286201; doi:10.1038/ncomms14061)
Supplement: Supplementary Information — Supplementary Figures 1-11 and Supplementary References [file ncomms14061-s16.pdf]

Supplementary Information

Supplementary Figures

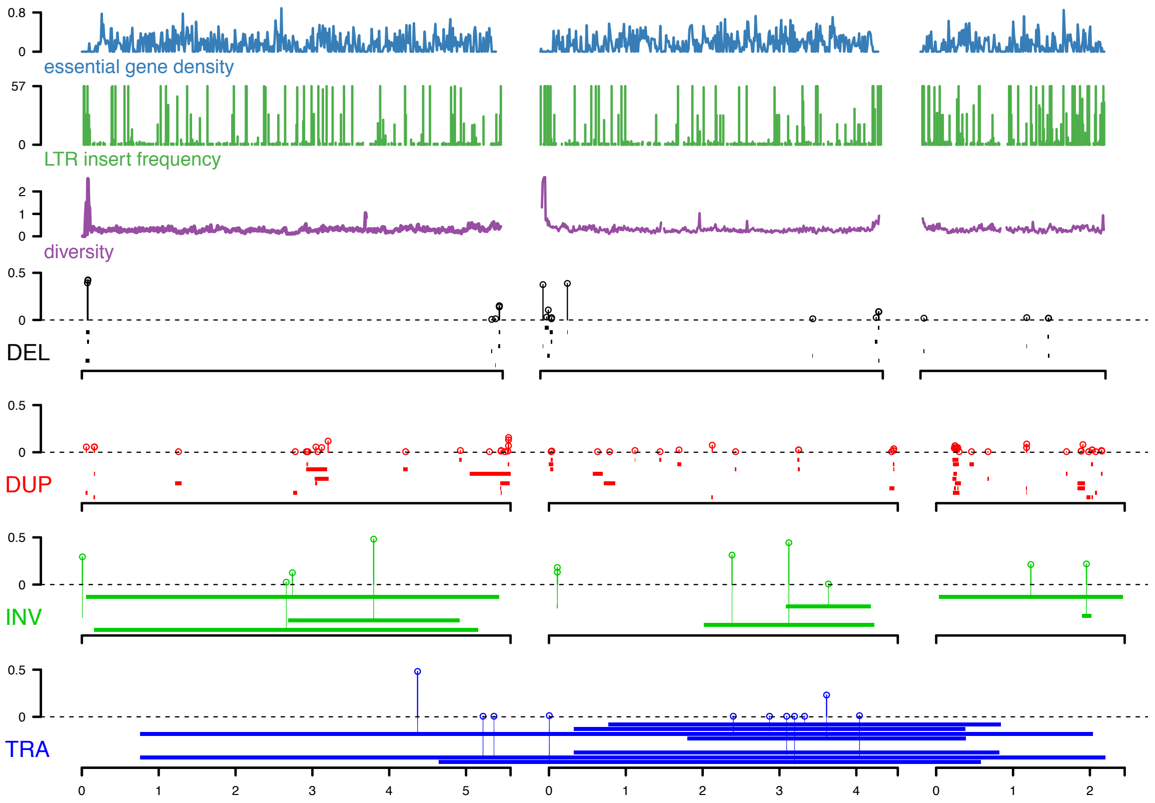

Supplementary Figure 1. Locations and minor allele frequencies of all structural variants in curated data set. Each of the three chromosomes is indicated by black bar, with scale (in megabases) at bottom. From top (same data as Fig 1): density of essential genes (blue), locations of *Tf*-type retrotransposons (green), and diversity (π, average pairwise diversity from SNPs, purple). Bar heights for deletions and duplications are proportional to minor allele frequency, the scale for retrotransposons is the frequency of the insertion in the 57 non-clonal strains. Diversity and retrotransposon were calculated from 57 non-clonal strains as described in Jeffares, et al. [1]. Below, we show different types of SVs: deletions (black), duplications (red), inversions (green) and translocations (blue). The vertical lines terminating with open circles above dotted lines emit from the mid-point of each SV and indicate the minor allele frequencies in the population of 161 strains.

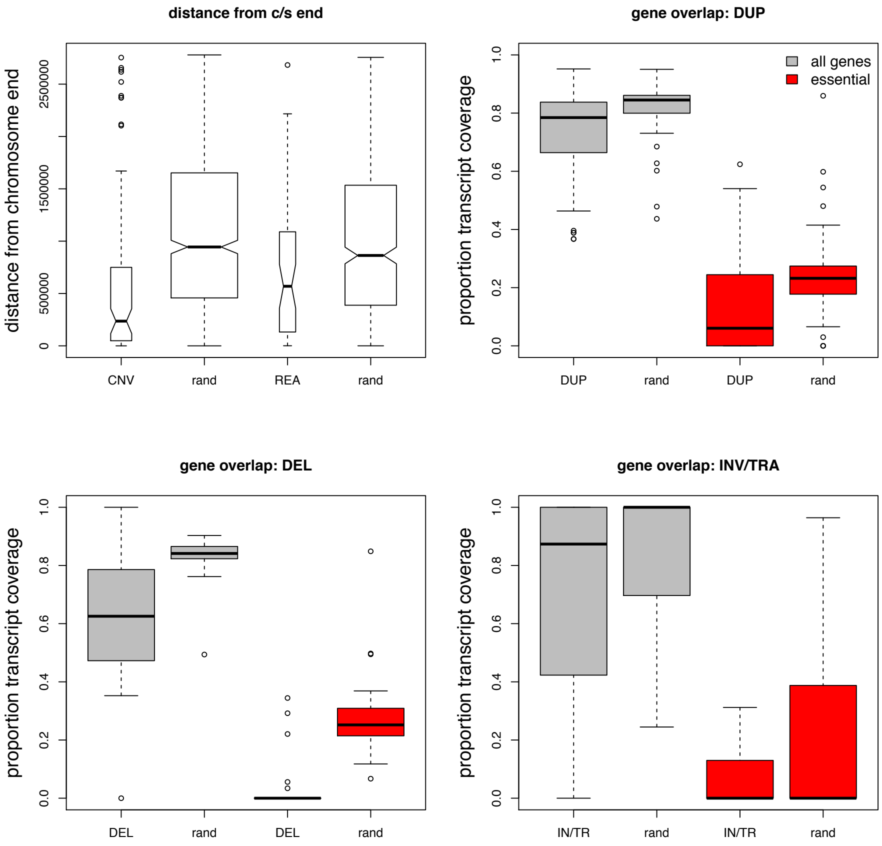

Supplementary Figure 2. Structural variations are biased towards chromosome ends and to low gene density regions. Top left panel, both CNVs and rearrangements are biased towards the ends of chromosomes. CNVs; median distance to chromosome ends 236 kb compared to chromosome- and size-matched random sites 944 kb, Wilcoxon rank sum test P = 1.3 x 10^{-11}, rearrangements median distance 569 kb *vs,* matched random 863 kb, Wilcoxon test P = 0.03). All other panels, calculated proportion of each duplication and deletion that contained all protein-coding or essential genes. Box plots show the distributions of these proportions for all genes (grey), and proportion of coverage by essential genes (red), compared to the null distribution (rand). All comparisons were significantly less than the null distributions (Wilcoxon rank sum test, P-values <1.6 x 10^{-4}). The same analysis was performed with the junctions of inversions and translocations, by calculating the transcript coverage in the region 500 bp up- and down-stream of the predicted start and end junctions. These rearrangements are slightly biased away from genes (P = 1.9x10^{-3}), but not significantly biased away from essential genes (P >0.05). The null distributions were determined by selecting 10 regions for each actual variant/junction that were the same size, and were placed in random positions on the same chromosome and calculating the gene coverage of these regions. Essential genes were those with the Fission Yeast Phenotype Ontology term defined as FYPO:0002061 (“inviable”) in PomBase.

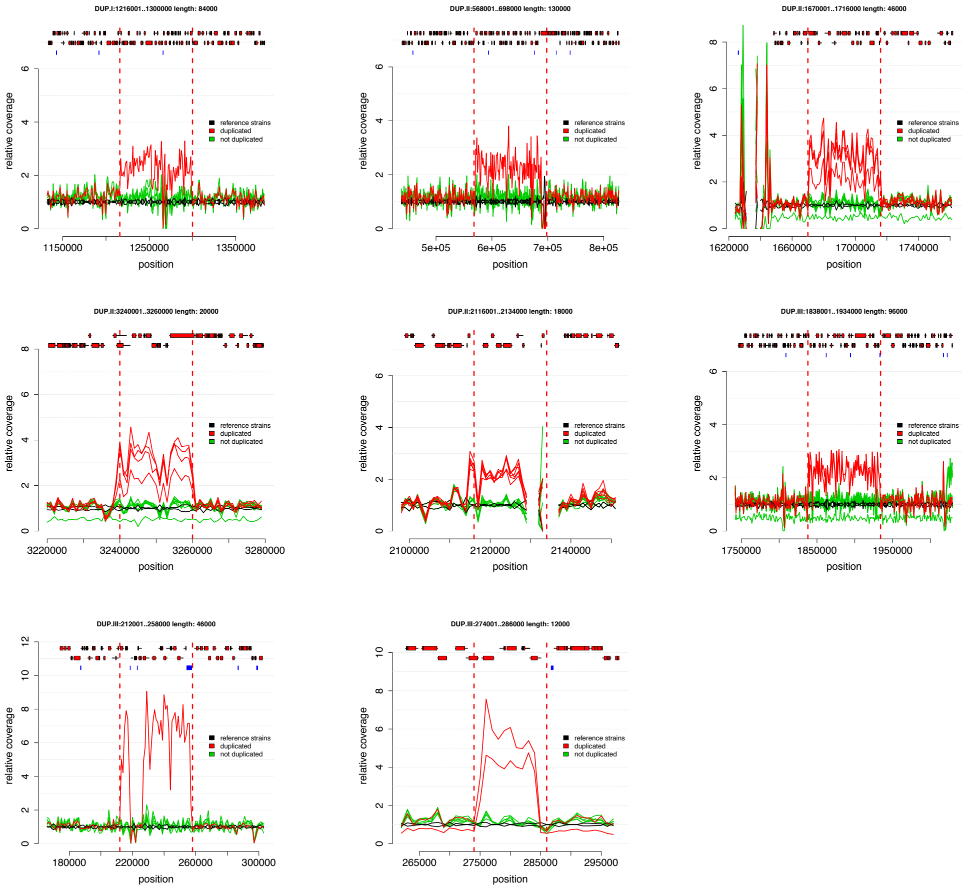

Supplementary Figure 3. Duplications that segregate within closely related strains. Plots show the average coverage in 1 kb non-overlapping windows for strains with a duplication (red) and all closely related strains without duplication (green); all these strains differ by <150 SNPs. The coverage of the two standard reference strains (h^+ and h^-) is shown in black. Top row, from left: variant DUP.I:1216001..1300000 (cluster 12, from Japan in 57), DUP.II:568001..698000 (cluster 12), DUP.II:1670001..1716000 (cluster 2, unknown origin), second row DUP.II:3240001..3260000 (cluster 2), DUP.II:2116001..2134000 (cluster 1, includes reference strain from French grapes in 1947), DUP.III:1838001..1934000 (cluster 2, various locations 1921-22). Bottom row: DUP.III:212001..258000 (cluster 6, Jamaica/USA), and DUP.III:274001..286000 (cluster 5, Sicily 1966). Genes are shown on top of plots with exons as red rectangles and retrotransposon LTRs as blue rectangles.

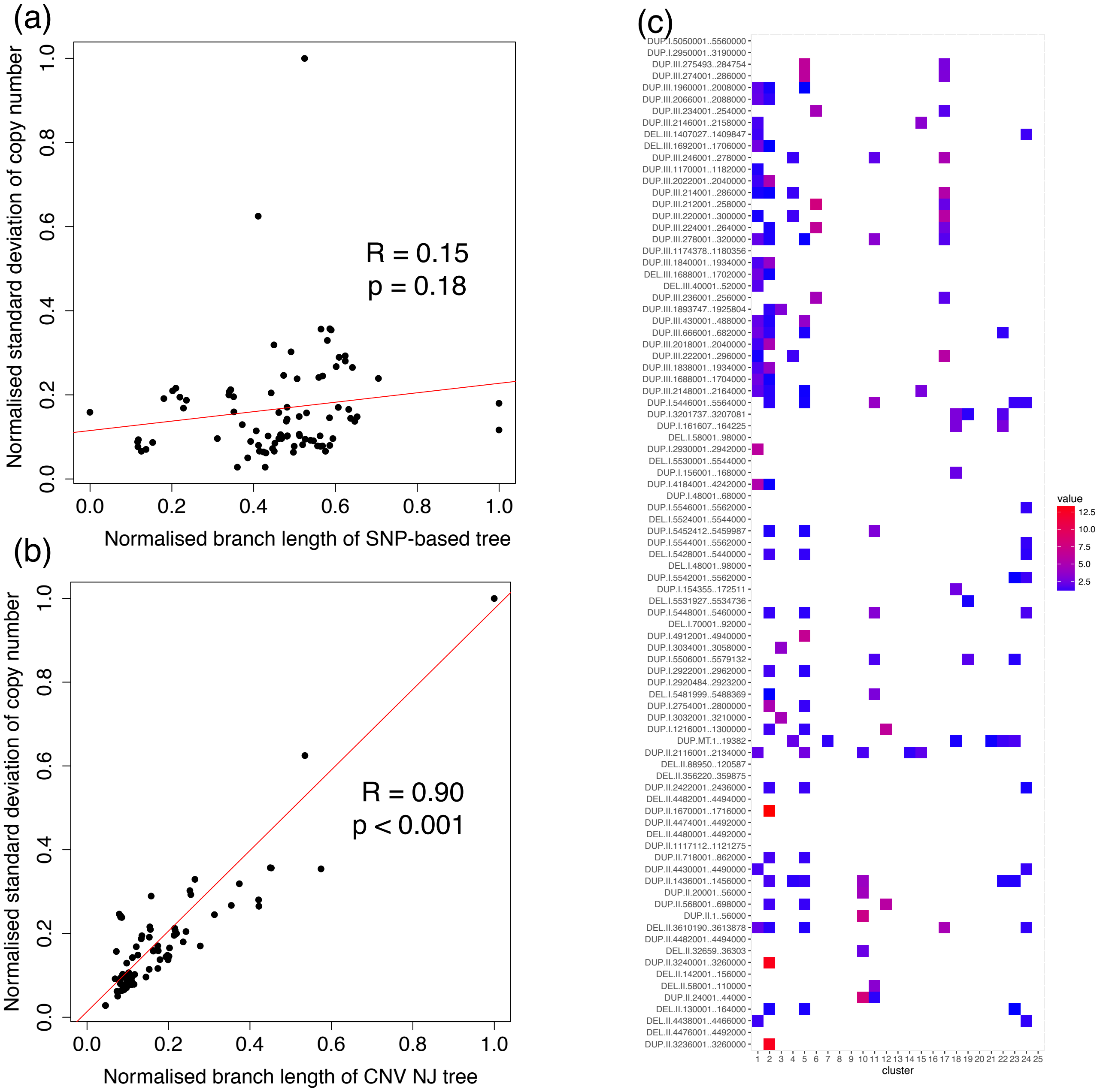

Supplementary Figure 4. Relative standard deviation of copy number variation within clusters. (a) Standard deviation of copy number for a CNV across the dataset is only weakly correlated with the total branch length of SNP-based phylogeny from the 20kb up- and down-stream phylogeny. **(b)** Standard deviation is highly correlated with the branch length of a CNV-based neighbour-joining tree. **(c)** The relative standard deviation of each CNV within each identified cluster of strains (<150 SNPs apart) relative to its change in rest of the dataset. For clarity, all relative standard deviations <1 are shown as white.

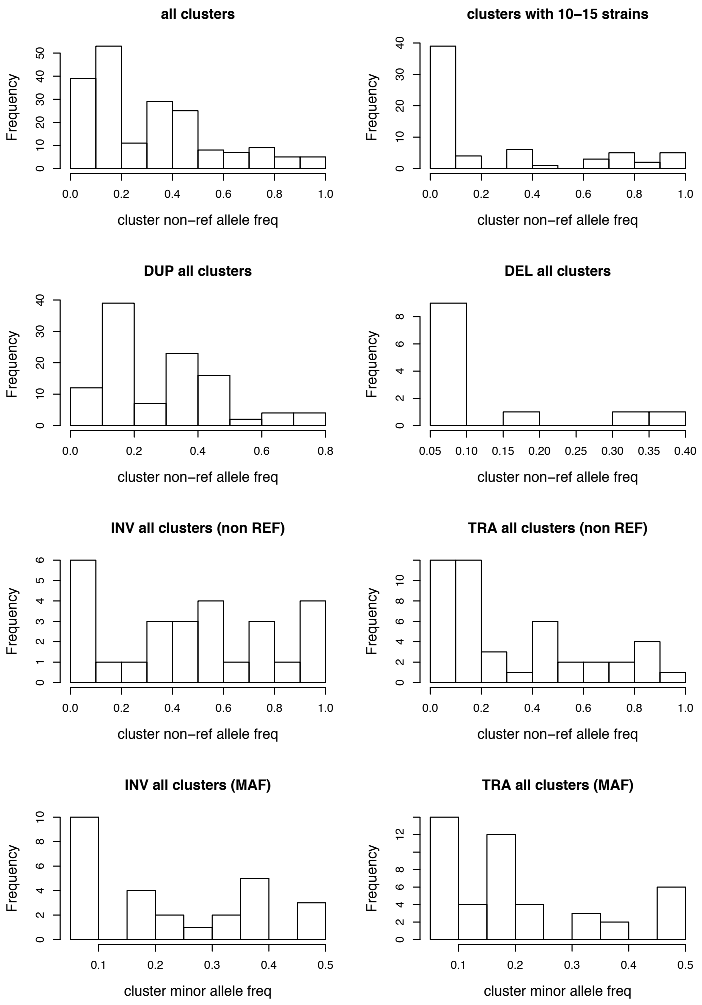

Supplementary Figure 5. Copy number variants are usually rare alleles within clonal populations. Clonal clusters, or clonal populations all differ by < 150 SNPs. In rows, from top left; we show the within-cluster frequency of the non-reference allele for all SVs, which is skewed to rare alleles. Limiting this analysis to cluster with 10 to 15 strains highlights the low frequency of non-reference alleles. Second row; CNVs (duplications and deletions) are skewed to rare alleles, because the non-reference allele is usually the derived allele. Third row; inversions and translocations are not skewed to the non-reference allele, but here non-reference alleles are not necessarily the derived allele. Bottom row; the *minor allele* of inversions and translocations, however, is skewed to rare alleles.

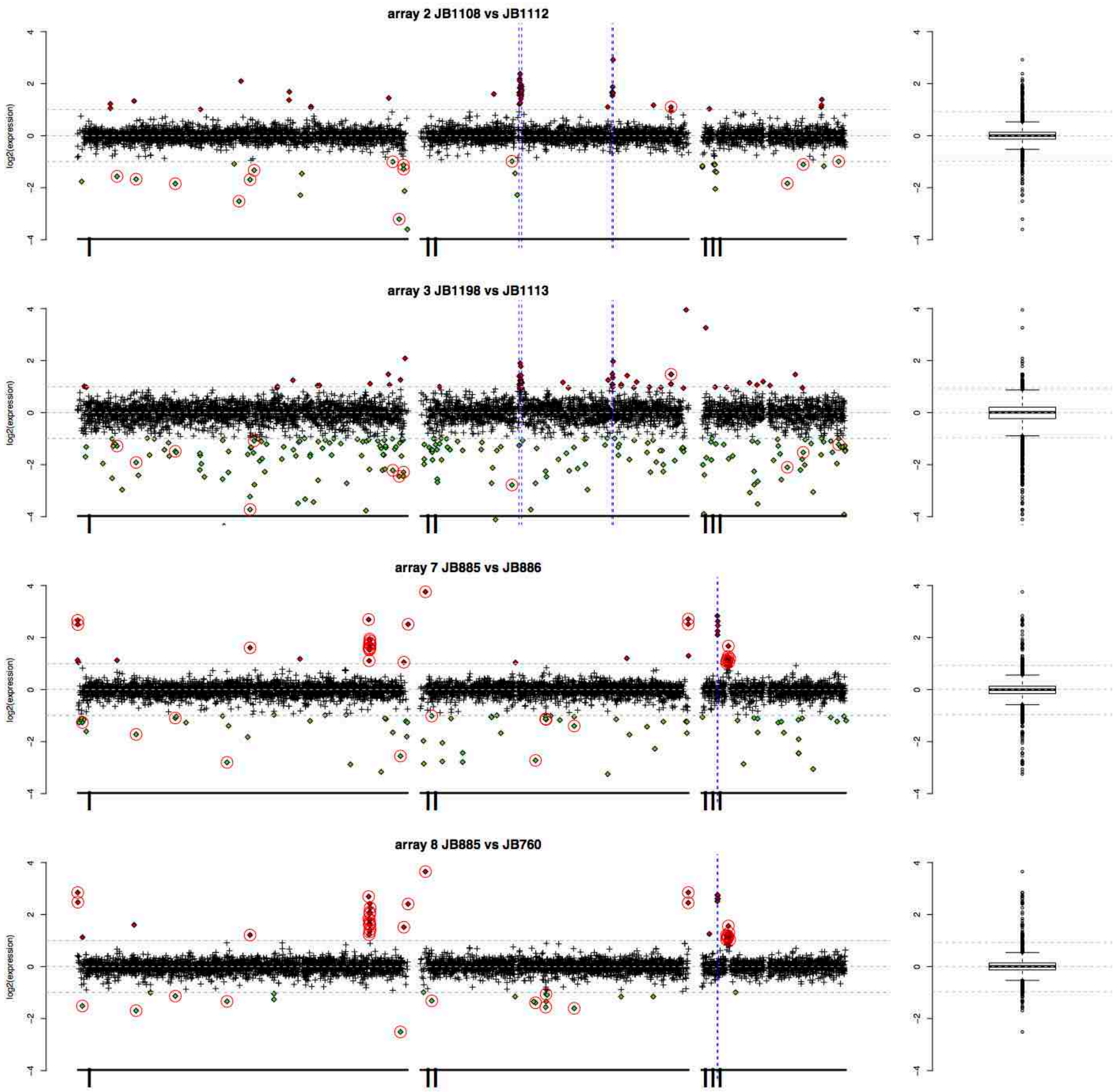

Supplementary Figure 6. Chromosome-scale view of gene expression changes. The relative gene expression levels (strain1/strain2) for arrays 2 and 3, and arrays 7 and 8 are shown with their positions on the three chromosomes. Filled circles indicates genes that we consider to be up-regulated (red) or repressed (green). Those highlighted with open red circles are consistently altered in both arrays (either 2+3, or 7+8). The blue lines show where the segregating duplications are. Box plots at right show the spread of data.

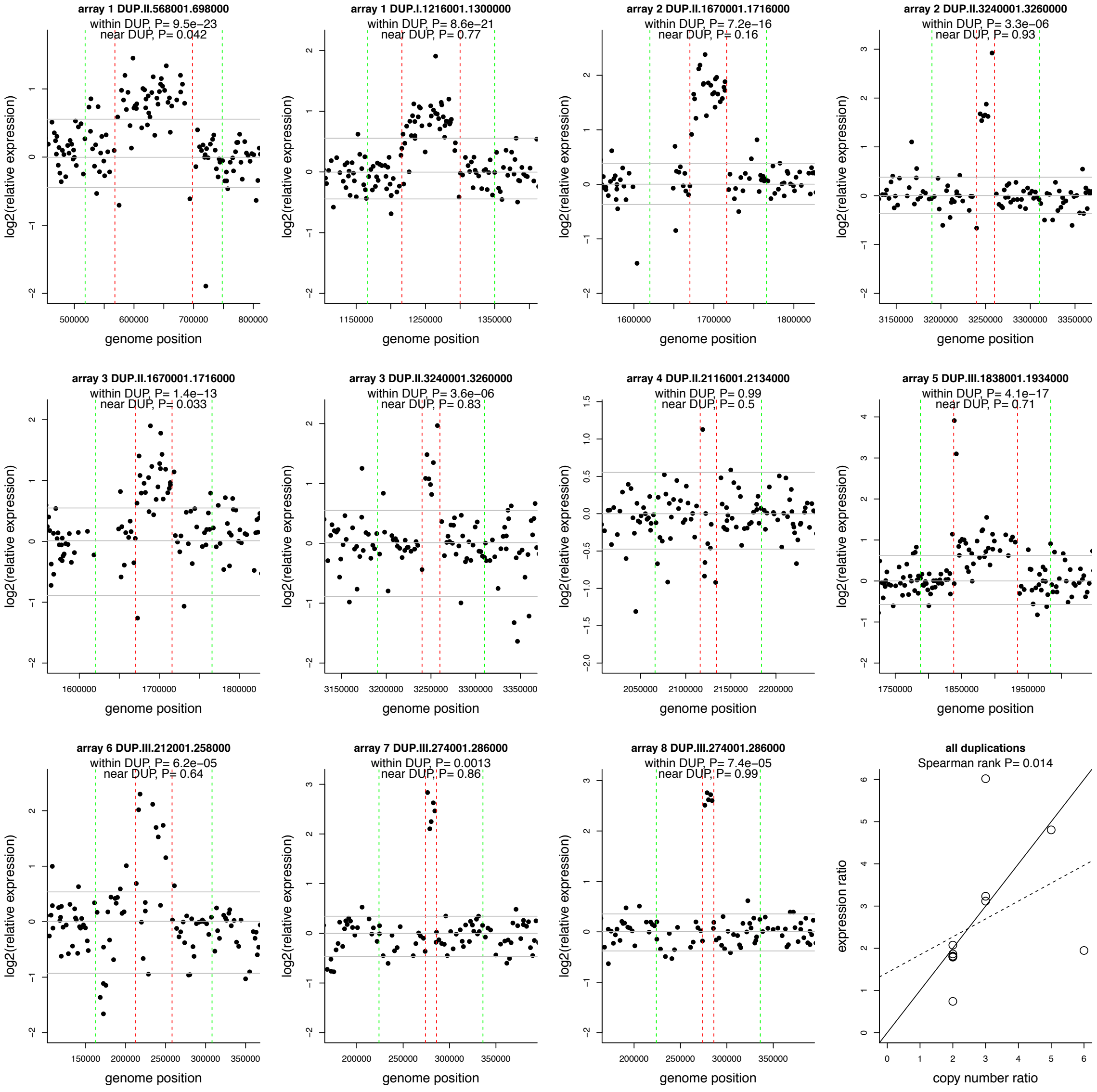

Supplementary Figure 7. No significant increase in gene expression immediately adjacent to duplications. For each duplication examined with DNA arrays, we show the relative expression (strain 1 *vs* strain 2) near the duplication. P-values show the support for the genes within the duplication (red vertical lines), or the 50 kb adjacent to the duplication (green vertical lines) being more highly expressed than all other genes in the chromosome (one-sided Wilcoxon rank sum tests). The grey horizontal lines show the 5th, 50th and 95th percentiles for gene expression data on the chromosome. The bottom right panel shows that the median increase in expression level within a duplication correlates with the increase in genomic copy number. The solid back line shows the expected increase for the 1:1 correspondence between genomic copy number and relative expression (the line $y=x$), and the dashed line shows the linear model for the data. Copy number and relative expression change are correlated (Spearman rank correlation $\rho = 0.71$ and $P = 0.014$).

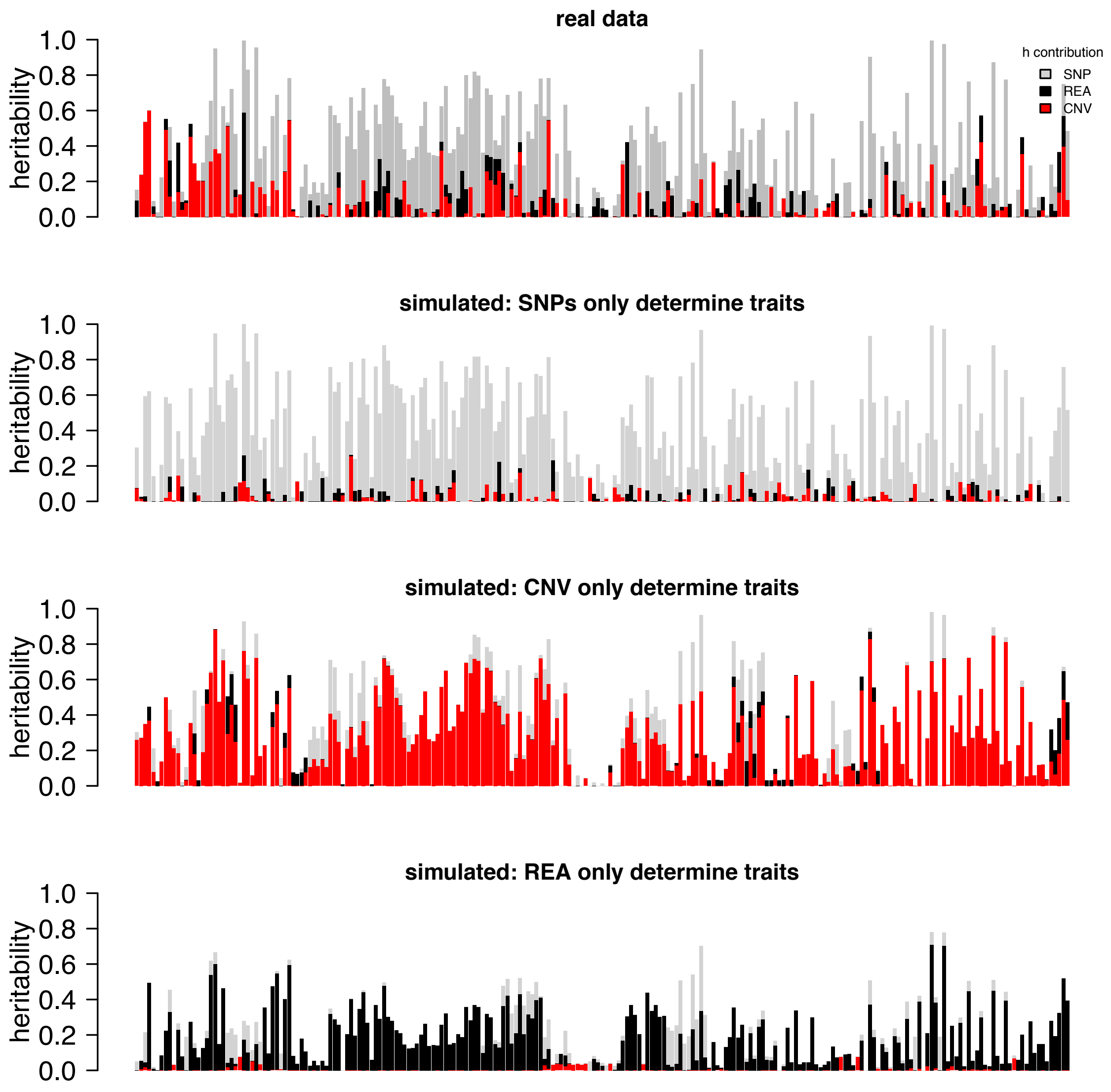

Supplementary Figure 8. Contributions of SNPs, CNVs and rearrangements to traits. Top panel: for 227 traits, we show the total heritability estimated by the combination of 243,289 SNPs (green), 87 CNVs (red), and 26 rearrangements (grey). We then simulated data that was entirely due to the effects of SNPs (second panel), entirely due to the effects of CNVs (next panel) or entirely due to the effects of rearrangements (lower). In the second panel (entirely due to the effects of SNPs), the contribution of CNVs and rearrangements are artefacts, but these are relatively minor. This analysis indicates that the estimates are not strongly affected by linkage.

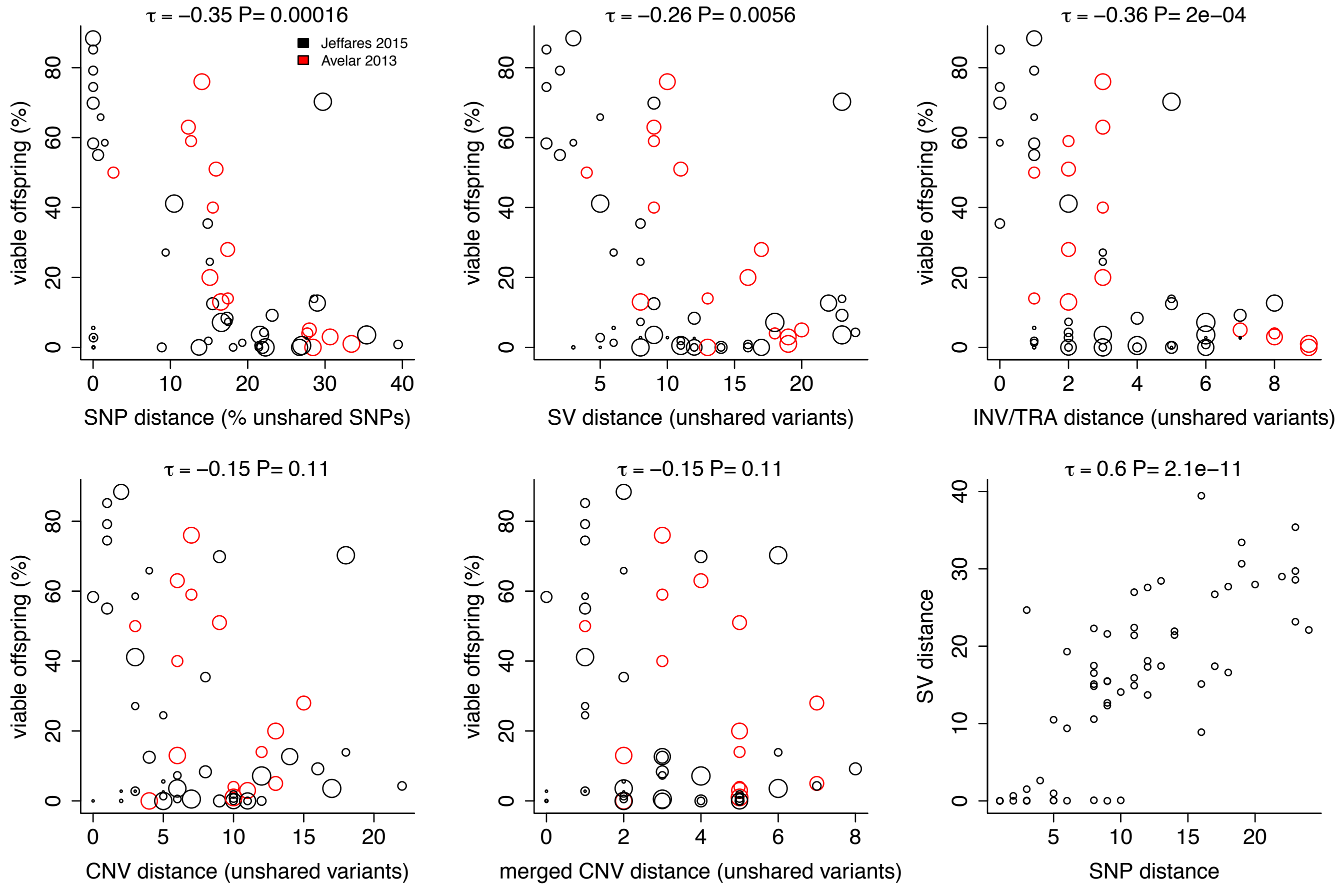

Supplementary Figure 9. Correlations between spore viability, parental SNP-genetic distance and parental SV-genetic distance. Spore viability was measured for 58 crosses in total, including data from both Jeffares, et al. [1] (black) and Avelar, et al. [2] (red), with each circle representing one cross. Clockwise from top left plots show correlation of spore viability with various measures of genetic distances between parents; SN distance, all SV distances, all rearrangement differences (inversions/translocations), unmerged CNV distances, merged CNV distances, and finally the relationship between SNP distance and SV distance. Unmerged CNV differences count any CNV as being different between parents when either start or end coordinates are more than 1 kb apart. Because this definition can cause us to count largely overlapping events as 'different', we also counted 'merged' differences where two CNVs were considered different only if their overlap was >50% of the total of both variants. This approach will exclude nested CNVs. CNV-genetic distance is not significantly correlated with viability in either case.

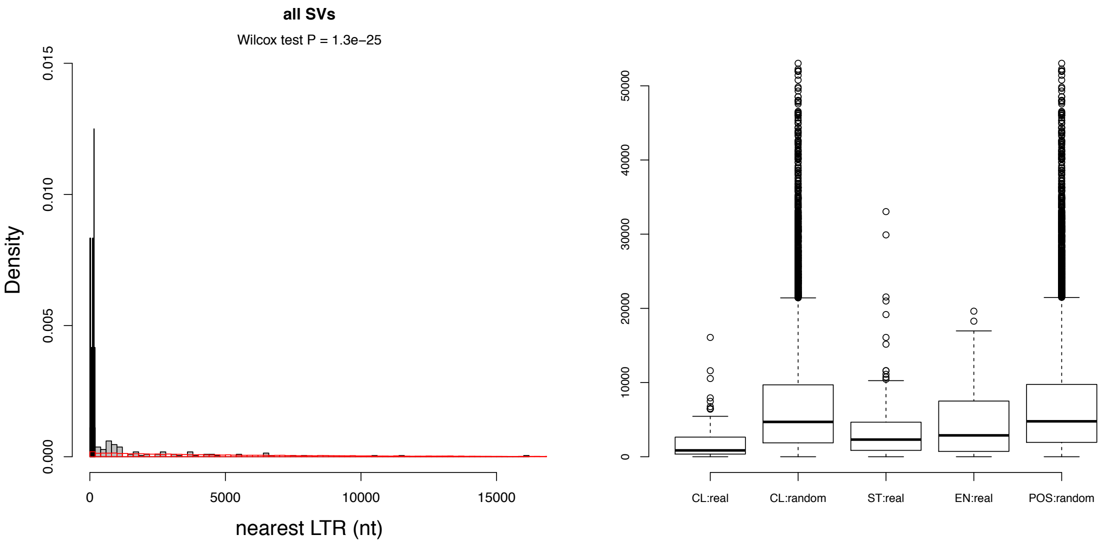

Supplementary Figure 10. SVs are enriched close to retrotransposon LTRs. For all SVs, we computed the closest distance of start or end coordinates to any LTR discovered previously [1]. As a control, we compute the closest distance of 10 random coordinates on the same chromosome. Left: the distributions of distances for real SVs (grey), those that are within 200nt (black) or random coordinates (red). Right: using the same analysis, we show the closest distance of real SVs (CL: real), and random coordinates (C: random). We also show that both start and end coordinates of SVs (ST:real, EN:real) are closer than random positions (POS:random).

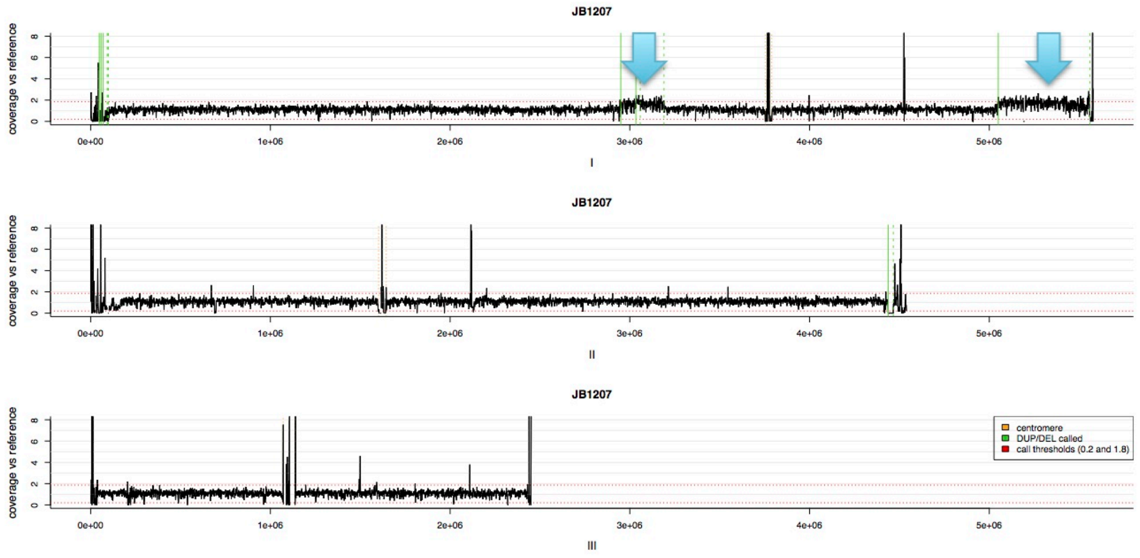

Supplementary Figure 11. Chromosome-scale read coverage plots for three chromosomes of strain JB1207. Coverage is calculated relative to the reference strain (JB22 in our collection). Two large duplications that did not satisfy the criteria used to detect CNVs with cn.MOPs are indicated with blue arrows (DUP.I:2950001..3190000, 240kb and DUP.I:5050001..5560000, 510kb).

Supplementary References

1 Jeffares, D. C. *et al.* The genomic and phenotypic diversity of Schizosaccharomyces pombe. *Nat Genet* **47**, 235-241, doi:10.1038/ng.3215 (2015).

2 Avelar, A. T., Perfeito, L., Gordo, I. & Ferreira, M. G. Genome architecture is a selectable trait that can be maintained by antagonistic pleiotropy. *Nat Commun* **4**, 2235, doi:10.1038/ncomms3235 (2013).
